# The prevalence of multimorbidity in primary care: a comparison of two definitions of multimorbidity with two different lists of chronic conditions in Singapore

**DOI:** 10.1186/s12889-021-11464-7

**Published:** 2021-07-16

**Authors:** Eng Sing Lee, Poay Sian Sabrina Lee, Ying Xie, Bridget L. Ryan, Martin Fortin, Moira Stewart

**Affiliations:** 1 Clinical Research Unit, National Healthcare Group Polyclinics, Singapore, Singapore; 2 Department of Epidemiology and Biostatistics, Schulich School of Medicine & Dentistry, Western University, 1151 Richmond St, London, ON N6A 5C1 Canada; 3 Centre for Studies in Family Medicine, Department of Family Medicine, Schulich School of Medicine & Dentistry, Western University, 1151 Richmond St, London, ON N6A 5C1 Canada; 4 Department of Family Medicine, Centre de Santé et de Services Sociaux de Chicoutimi, Unité de médecine de famille, University of Sherbrooke, 305, rue St-Vallier, Chicoutimi, QC G7H 5H6 Canada

**Keywords:** Multimorbidity, Prevalence, Age, Sex, Ethnicity, Primary care

## Abstract

**Background:**

The prevalence of multimorbidity varies widely due to the lack of consensus in defining multimorbidity. This study aimed to measure the prevalence of multimorbidity in a primary care setting using two definitions of multimorbidity with two different lists of chronic conditions.

**Methods:**

We conducted a cross-sectional study of 787,446 patients, aged 0 to 99 years, who consulted a family physician between July 2015 to June 2016. Multimorbidity was defined as ‘two or more’ (MM2+) or ‘three or more’ (MM3+) chronic conditions using the Fortin list and Chronic Disease Management Program (CDMP) list of chronic conditions. Crude and standardised prevalence rates were reported, and the corresponding age, sex or ethnic-stratified standardised prevalence rates were adjusted to the local population census.

**Results:**

The number of patients with multimorbidity increased with age. Age-sex-ethnicity standardised prevalence rates of multimorbidity using MM2+ and MM3+ for Fortin list (25.9, 17.2%) were higher than those for CDMP list (22.0%; 12.4%). Sex-stratified, age-ethnicity standardised prevalence rates for MM2+ and MM3+ were consistently higher in males compared to females for both lists. Chinese and Indians have the highest standardised prevalence rates among the four ethnicities using MM2+ and MM3+ respectively.

**Conclusions:**

MM3+ was better at identifying a smaller number of patients with multimorbidity requiring higher needs compared to MM2+. Using the Fortin list seemed more appropriate than the CDMP list because the chronic conditions in Fortin’s list were more commonly seen in primary care. A consistent definition of multimorbidity will help researchers and clinicians to understand the epidemiology of multimorbidity better.

**Supplementary Information:**

The online version contains supplementary material available at 10.1186/s12889-021-11464-7.

## Background

By 2050, the world’s population aged 60 years and older is estimated to be 2 billion people [1]. Not only does a longer life expectancy increase the chance of developing one chronic condition, but the possibility of having multiple chronic conditions (i.e., multimorbidity) also increases. Multimorbidity poses a major challenge to the healthcare systems as increased number of people have multimorbidity with age [1].

Estimates for the prevalence of multimorbidity in primary care vary widely (12.9 to 95.1%) due to the inconsistencies in the definition of multimorbidity [2]. The measurement of multimorbidity based on counting number of chronic conditions depends on five components that are closely related to the definition of the entity: a) the types of conditions selected to form the multimorbidity list; b) the total number of conditions considered in the multimorbidity list; c) the cut-off threshold for the number of chronic conditions used to define multimorbidity; d) the data sources of the conditions; and e) the reference population being measured [3–5]. The lack of reporting or consensus on the five components have made comparisons between prevalence rates found in different studies difficult, preventing reliable estimations of disease burden and hinder resource distribution for effective disease management. Our recent work has proposed that an ideal operational definition of multimorbidity should comprise at least 12 chronic diseases, each with high burden and clinically relevant to the particular healthcare setting of interest [6].

The types of conditions, number of conditions and the cut-off thresholds used are the more contentious components. There are no fixed conditions used for the multimorbidity list; the number of conditions used in multimorbidity prevalence studies range from 4 to 147 [7]. Fortin et al. suggested a list of 20 conditions for multimorbidity that were prevalent or had high patient impact [8]. However, a different list of 20 conditions under the Chronic Disease Management Programme (CDMP) list from the Ministry of Health of Singapore (MOH) identified for government subsidy locally may also be suitable [9]. Although the World Health Organization defines multimorbidity as the co-occurrence of two or more chronic conditions in an individual [10], Fortin et al. suggested that studies should include two operational definitions of multimorbidity, i.e., for two or more chronic conditions and three or more chronic conditions [3].

This study sought to examine the prevalence of multimorbidity in the Singapore primary care population using electronic medical records. The objectives of this study were to: (1) describe the epidemiology of chronic conditions using two lists of conditions (International list: Fortin and Local list: CDMP) to define multimorbidity; (2) determine the crude and standardised prevalence rates of multimorbidity based on the two different lists of chronic conditions with two operational definitions of multimorbidity based on the two commonly reported cut-off thresholds (2+ and 3+ chronic conditions); and (3) determine the differences in the standardised prevalence rates among the different age, sex and ethnic groups.

## Methods

This was a cross-sectional study of patients aged 0 to 99 years who consulted a family physician at any of the nine National Healthcare Group Polyclinics (NHGP) in Singapore between 1st July 2015 and 30th June 2016 and who had an ICD-10 (International Classification of Diseases, Tenth Revision) diagnosis code. A polyclinic is a government-funded primary care centre that offers a one-stop centre including chronic disease management [11]. Data were obtained from the electronic medical records. The denominator was all patients who had consulted a family physician at least once in the stipulated time frame.

The numerator was the number of patients that had multimorbidity. The numerator was chosen with the following considerations. Firstly, we adopted the definition of chronicity of a disease as lasting at least six months, having a documented pattern of recurrence or deterioration, and having an impact on an individual’s quality of life [12]. Secondly, we used two different lists of chronic conditions to measure multimorbidity – a local list (CDMP list – Additional file 1) and an international list (Fortin list – Additional file 2). Thirdly, we used two cut-off thresholds – ‘two or more’ chronic conditions (MM2+) and ‘three or more’ chronic conditions (MM3+). As such, there were four numerators: the operational definition of MM2+ using the CDMP list (CDMP MM2+), MM3+ using the CDMP list (CDMP MM3+), MM2+ using the Fortin list (Fortin MM2+), and MM3+ using the Fortin list (Fortin MM3+).

We matched all the NHGP ICD-10 diagnosis codes classified as a chronic condition to both lists because the CDMP list was based on diagnoses stipulated on the MOH website and the Fortin list was based on ICPC-2 (International Classification of Primary Care – 2nd edition). Four NHGP senior family physicians were consulted on the appropriateness of the matched ICD-10 codes to the two lists. They unanimously agreed on the appropriateness of the matching of all CDMP conditions to the ICD-10 diagnoses but found only 19 of the 20 Fortin conditions to be appropriately matched to the ICD-10 diagnosis codes. ‘Back pain’ in the Fortin list was not matched with the NHGP ICD-10 codes because the condition was usually coded as an acute rather than a chronic condition in NHGP. Ultimately, there were 26 ICD-10 codes and 39 ICD-10 codes matched to the CDMP and Fortin lists respectively (described in Additional file 1 and Additional file 2).

IBM SPSS version 21 and Microsoft Office Excel 2016 were used for all statistical calculations and analyses. We described the means for continuous variables with their respective standard deviations and proportions with their respective confidence intervals where appropriate. For corresponding age/sex/ethnic stratified standardised prevalence rates, we provided the weighted average of prevalence rate where the weights were the proportions of persons according to the 2016 Singapore Population [13, 14]. Confidence intervals of 95% were calculated using the Poisson approximation around the standardised rates among the different age, sex and ethnic groups. We considered no overlap of the 95% confidence intervals for the standardised prevalence rates among the different groups as statistically significant. For multiple comparisons, Bonferroni adjustment was applied.

## Results

### Epidemiology of chronic conditions using two lists of conditions (CDMP and Fortin)

This study included 787,446 unique patients from nine NHG Polyclinics. Table 1 shows the demographic characteristics of the study population compared to the national Singaporean population. The majority of the patients in this study were in the 45–64 years age group (32.0%). The Chinese formed the majority of the study population (68.2%), followed by Malays (16.2%), Indians (10.0%), and Others (5.6%). The mean age of Chinese patients was 47.1 years (±23.3) while the mean ages of Malay, Indian and Other patients were lower at 35.1 years (±22.1), 39.7 years (±21.1) and 37.1 (±19.1) respectively. There were slightly more females (50.9%) than males (49.1%). The mean age of the female patients was older at 45.3 (±23.2) years compared to the male patients at 42.4 (±23.2) years.

**Table 1:** Demographic characteristics of the patients in the study (*N* = 787,446)

	Number of patients (N)	Percentage (%)	National Proportion 2016 (%)	Age (years) Mean (SD^b^)	Number of CDMP^a^ Conditions, Mean (SD^b^)	Number of Fortin Conditions, Mean (SD^b^)
AGE						
0–24 years	201,839	25.6	26.9	12.9 (8.1)	0.0 (0.2)	0.1 (0.3)
25–44 years	165,212	21.0	30.5	34.0 (6.0)	0.2 (0.6)	0.4 (0.8)
45–64 years	252,206	32.0	29.3	55.4 (5.5)	1.3 (1.3)	1.7 (1.6)
65–99 years	168,189	21.4	13.3	73.5 (7.0)	2.4 (1.5)	3.0 (1.8)
ETHNICITY						
Chinese	537,234	68.2	74.3	47.1 (23.3)	1.1 (1.4)	1.4 (1.7)
Malay	127,501	16.2	13.4	35.1 (22.1)	0.8 (1.3)	1.0 (1.6)
Indian	78,452	10.0	9.1	39.7 (21.1)	0.9 (1.4)	1.2 (1.8)
Others	44,259	5.6	3.2	37.1 (19.1)	0.6 (1.1)	0.8 (1.4)
SEX						
Female	400,965	50.9	51.2	45.3 (23.2)	1.0 (1.3)	1.3 (1.7)
Male	386,481	49.1	48.8	42.4 (23.2)	1.0 (1.4)	1.3 (1.7)

^a^ *CDMP* Chronic Disease Management Programme, ^b^ *SD* Standard deviation

The mean number of chronic conditions increased with age. In Table 1, we observed that patients under 45 years old had fewer chronic conditions for both lists. On the other hand, the oldest group aged 65 years and above had the highest mean number of chronic conditions; mean of 2.4 (±1.5) and 3.0 (±1.8) chronic conditions when the CDMP and Fortin lists were used respectively. The Chinese had the highest mean number of chronic conditions (CDMP: 1.1, Fortin: 1.4), followed by the Indians (CDMP: 0.9, Fortin: 1.2), then Malays (CDMP: 0.8, Fortin: 1.0), and lastly Others (CDMP: 0.6, Fortin: 0.8).

Table 2 reports the patient count and crude prevalence rates of the individual chronic conditions for both lists. The three most prevalent conditions in both lists were ‘hyperlipidaemia’, ‘hypertension’ and ‘diabetes’, with prevalence rates above 10.0%. Additionally, the Fortin list also identified a fourth condition, ‘Arthritis &/or Rheumatoid Arthritis’, which also had a prevalence rate above 10.0%. There were nine chronic conditions with prevalence rates above 1.0% in the CDMP list compared to 16 chronic conditions in the Fortin list.

**Table 2:** Crude prevalence rates of single chronic conditions from CDMP and Fortin lists

Rank	CDMP^a^ Conditions	Patient Count	%	Fortin Conditions	Patient Count	%
1	Hyperlipidaemia	257,114	32.7	Hyperlipidaemia	257,114	32.7
2	Hypertension	221,760	28.2	Hypertension	221,760	28.2
3	Diabetes	125,058	15.9	Diabetes	124,954	15.9
4	Ischaemic Heart Disease	36,401	4.6	Arthritis &/or Rheumatoid arthritis	100,838	12.8
5	Asthma	28,778	3.7	Obesity	48,893	6.2
6	Chronic Kidney Disease	21,638	2.7	Cardiovascular disease (Angina, Myocardial infarction, Atrial fibrillation, poor circulation of lower limbs)	43,559	5.5
7	Stroke	19,808	2.5	Asthma, COPD^b^, or Chronic bronchitis	32,611	4.1
8	Osteoarthritis	18,378	2.3	Chronic hepatitis	25,918	3.3
9	Benign Prostate Hypertrophy	13,031	1.7	Stroke and Transient Ischaemic Attack	23,628	3.0
10	Osteoporosis	7283	0.9	Stomach problem (reflux, heartburn, or gastric ulcer)	22,233	2.8
11	Anxiety	6085	0.8	Kidney disease or failure	22,221	2.8
12	Chronic Obstructive Pulmonary Disease (COPD)	5080	0.6	Thyroid disorder	20,781	2.6
13	Dementia	3571	0.5	Heart failure (including valve problem or replacement)	20,538	2.6
14	Schizophrenia	2889	0.4	Depression or anxiety	14,910	1.9
15	Epilepsy	2734	0.3	Chronic urinary problem	13,031	1.7
16	Rheumatoid Arthritis	2010	0.3	Any Cancer in the last 5 years	7940	1.0
17	Parkinson’s	1900	0.2	Osteoporosis	7283	0.9
18	Major Depression	1700	0.2	Dementia or Alzheimer’s disease	3571	0.5
19	Psoriasis	651	0.1	Colon problem (irritable bowel)	1571	0.2
20	Bipolar Disorder	51	0.0			

^a^ *CDMP* Chronic Disease Management Programme, ^b^ *COPD* Chronic obstructive pulmonary disease

**Fig. 1:**
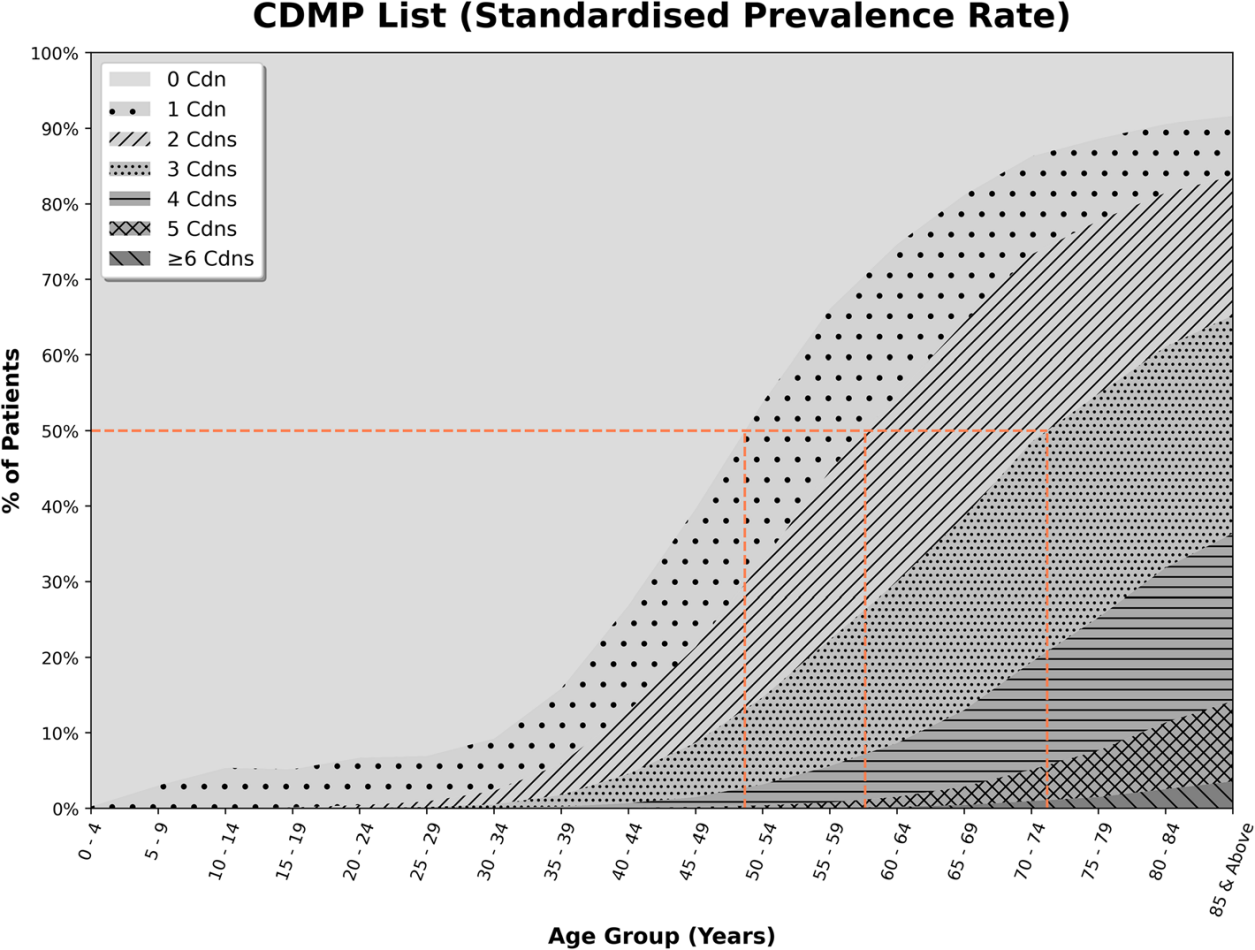
Number of chronic conditions by age-group (CDMP list)

**Fig. 2:**
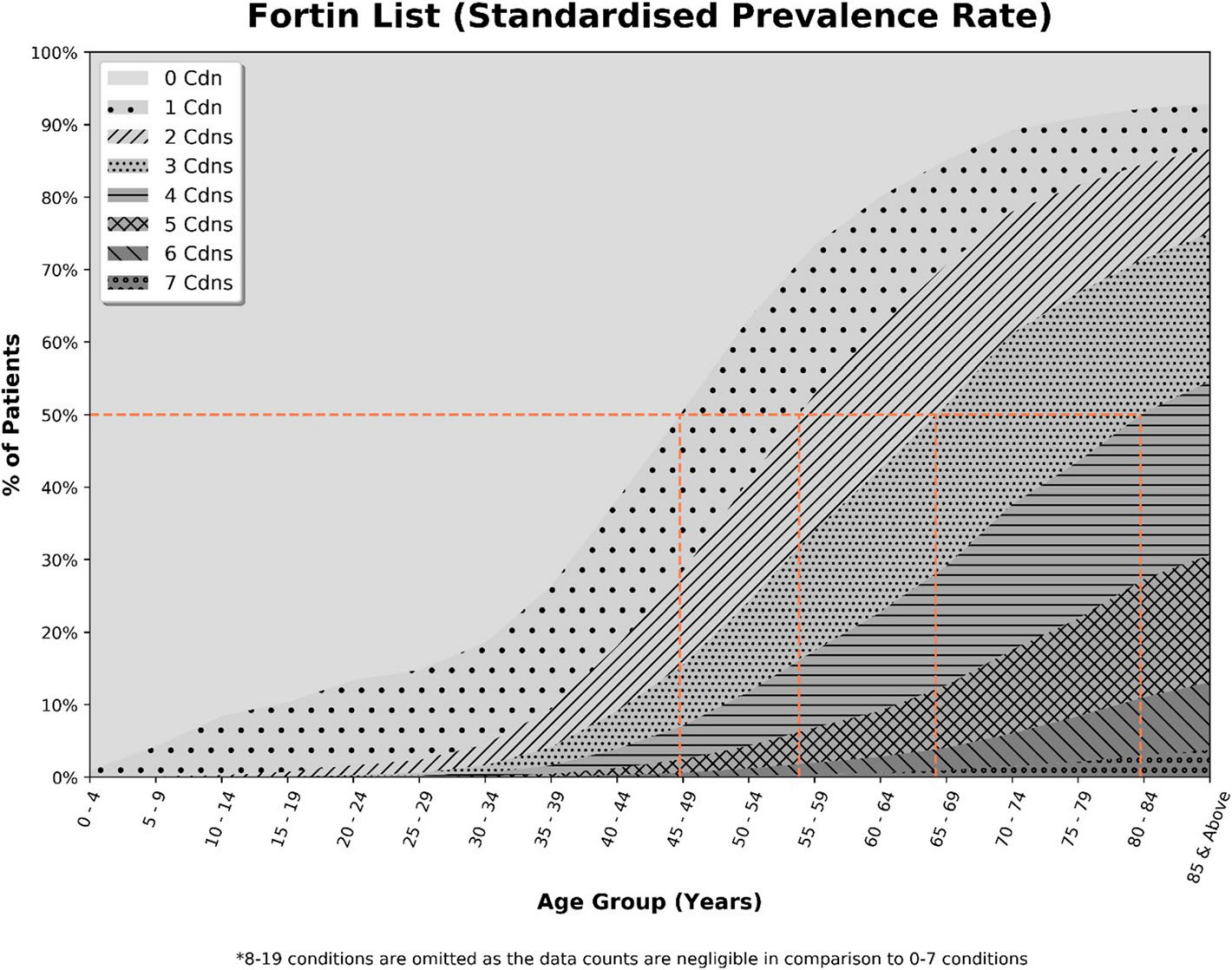
Number of chronic conditions by age-group (Fortin list)

Figures 1 and 2 report the percentage of patients with chronic conditions based on the standardized prevalence rate for sex and ethnicity, stratified by age. Both figures showed that the proportion of patients with chronic conditions increased with the advancement of age. The CDMP list (Fig. 1) showed that 50% of the population in primary care had one chronic condition in their late 40s, two chronic conditions in their late 50s and three chronic conditions in their early 70s. The Fortin list (Fig. 2) showed that 50% of the population in primary care had chronic condition(s) at earlier ages i.e., one chronic condition in their early 40s, two chronic conditions in their early 50s, three chronic conditions in their early 60s, and four chronic conditions in their late 70s.

### Prevalence rates of multimorbidity based on two different lists with two operational definitions

The crude prevalence rates of multimorbidity in descending order were MM2+ Fortin at 33.9%, MM2+ CDMP at 29.7%, MM3 + Fortin at 23.7%, and MM3+ CDMP at 17.7%. The standardised prevalence rates of multimorbidity were of the same order but with a smaller magnitude after adjusting for age, ethnicity and sex. They were 25.9% (CI 25.8, 26.0), 22.0% (CI 21.9, 22.1), 17.2% (CI 17.2, 17.3), and 12.4% (CI 12.3, 12.5) respectively.

### Differences in the standardized prevalence rates among the different age, sex and ethnic groups

The standardised prevalence rates of multimorbidity increased with age as shown in Table 3. There were statistically significant differences among the standardised prevalence rates of multimorbidity among all four age groups.

**Table 3:** Standardized Prevalence Rates of Multimorbidity – prevalence rates are standardised for sex and ethnicity

	0–24 years		25–44 years		45–64 years		65–99 years	
	CPR^d^, %	ASE SPR^a^, % (CI^c^)	CPR^d^, %	ASE SPR^a^ % (CI^c^)	CPR^d^, %	ASE SPR^a^, % (CI^c^)	CPR^d^, %	ASE SPR^a^, % (CI^c^)
CDMP^b^ MM^e^2^+^	0.16	0.13 (0.12, 0.15)	5.3	5.8 (5.6, 5.9)	40.6	37.8 (37.6, 38.1)	72.7	72.5 (72.0, 72.9)
CDMP^b^ MM^e^3^+^	0.02	0.01 (0.01, 0.02)	1.7	1.9 (1.8, 2.0)	21.0	19.1 (18.9, 19.3)	49.9	49.7 (49.4, 50.0)
Fortin MM^e^2^+^	0.73	0.61 (0.58, 0.65)	8.1	8.9 (8.7, 9.0)	48.2	45.3 (45.1, 45.6)	77.5	77.3 (76.9, 77.7)
Fortin MM^e^3^+^	0.11	0.08 0.07, 0.10)	3.7	4.0 (3.9, 4.1)	30.9	28.5 (28.3, 28.7)	61.1	60.9 (60.5, 61.2)

^a^ *ASE SPR* Age-stratified, sex-and-ethnicity standardized prevalence rate, ^b^ *CDMP* Chronic Disease Management Programme, ^c^ *CI* Confidence interval, ^d^ *CPR* Crude prevalence rate, ^e^ *MM* Multimorbidity

The standardised prevalence rates of multimorbidity were higher for male patients when compared to female patients for both the CDMP and Fortin lists using both definitions of MM2+ and MM3+ (Table 4). The differences were statistically significant.

**Table 4:** Standardized Prevalence Rates – prevalence rates are standardised for age and ethnicity

	Female		Male	
	CPR^d^, %	SAE SPR^a^, % (CI^c^)	CPR^d^, %	SAE SPR^a^, % (CI^c^)
CDMP^b^ MM^e^2^+^	29.4	20.5 (20.4, 20.6)	29.9	23.5 (23.4, 23.7)
CDMP^b^ MM^e^3^+^	16.5	11.2 (11.1, 11. 3)	18.7	13.7 (13.6, 13.8)
Fortin MM^e^2^+^	34.6	25.0 (24.8, 25.1)	33.2	26.8 (26.7, 27.0)
Fortin MM^e^3^+^	24.0	16.5 (16.4, 16.6)	23.4	18.0 (17.9, 18.1)

^a^ *SAE SPR* Sex-stratified, age-and-ethnicity standardized prevalence rate, ^b^ *CDMP* Chronic Disease Management Programme, ^c^ *CI* Confidence interval, ^d^ *CPR* Crude prevalence rate, ^e^ *MM* Multimorbidity

Using the CDMP list and the MM2+ definition, the standardised prevalence rates of multimorbidity for the different ethnic groups in descending order were Chinese, Indians, Malays and Others (Table 5). For the MM3+ definition, the Indian ethnic group had the highest standardised prevalence rate of multimorbidity followed by Malays, Chinese, and the Others. The ranking order of the ethnic groups based on the standardized prevalence rates of the Fortin list was consistent for both MM2+ and MM3+ definitions. They were Chinese, followed by Indians, then Malays and Others. All the differences for both lists were statistically significant except for the standardised prevalence rates for Chinese (12.5% CI 12.4, 12.6) and Malays (12.6% CI 12.4, 12.8) using the CDMP MM3+ definition, and the standardised prevalence rates for Chinese (17.5% CI 17.4, 17.6) and Indians (17.5% CI 17.3, 17.8) using the Fortin MM3+ definition.

**Table 5:** Standardized Prevalence Rates – prevalence rates are standardised for age and sex

	Chinese		Malay		Indian		Others	
	CPR^d^, %	EAS SPR^a^, % (CI^c^)	CPR^d^, %	EAS SPR^a^, % (CI^c^)	CPR^d^, %	EAS SPR^a^, % (CI^c^)	CPR^d^, %	EAS SPR^a^, % (CI^c^)
CDMP^b^ MM^e^2^+^	33.0	22.7 (22.6, 22.8)	21.8	20.3 (20.0, 20.6)	27.2	21.2 (21.0, 21.5)	16.8	14.4 (14.1, 14.8)
CDMP^b^ MM^e^3^+^	19.3	12.5 (12.4, 12.6)	14.0	12.6 (12.4, 12.8)	17.7	13.1 (12.9, 13.4)	9.6	7.7 (7.4, 7.9)
Fortin MM2^+^	37.6	26.8 (26.7, 26.9)	24.9	23.5 (23.2, 23.8)	31.1	24.9 (24.6, 25.2)	19.7	17.3 (16.9, 17.7)
Fortin MM3^+^	26.0	17.5 (17.4, 17.6)	18.2	16.8 (16.6, 17.0)	23.0	17.5 (17.3, 17.8)	13.3	11.1 (10.8, 11.4)

^a^ *EAS SPR* Ethnicity-stratified, age-and-sex standardized prevalence rate, ^b^ *CDMP* Chronic Disease Management Programme, ^c^ *CI* Confidence interval, ^d^ *CPR* Crude prevalence rate, ^e^ *MM* Multimorbidity

## Discussion

### Summary of main findings

The crude prevalence rates of multimorbidity across all age groups ranged from 17.7 to 33.9%. The standardised prevalence rate ranged from 12.4% (CI 12.3, 12.5) to 25.9% (CI 25.8, 26.0) with MM3 + CDMP having the lowest standardised prevalence rate and MM2+ Fortin having the highest. Our study showed that using different definitions and lists of conditions for multimorbidity measurement resulted in different prevalence rates which were statistically different from each other when we adjusted for age, sex, and ethnicity for the same population. Whichever lists that we used, we found that prevalence rates of multimorbidity increased with age, and males had a statistically significantly higher prevalence rate of multimorbidity compared to females. There were also differences in prevalence rates of multimorbidity in the different ethnic groups, but the differences were dependent on operational definitions of multimorbidity.

### Comparison with existing literature

Our standardised prevalence rates using MM2+ (22.0% for CDMP; 25.9% for Fortin) were higher than that of MM3+ (12.4% for CDMP; 17.2% for Fortin). This is consistent with another study that reported the age-standardised prevalence rate of two or more chronic conditions to be higher when compared with three or more chronic conditions [15] (26.5 and 10.2% respectively using Canada’s 1991 population as the standard population).

There were six multimorbidity prevalence studies conducted using the definition of MM2+ in Singapore. None of the studies described clearly how the chronic conditions on the lists used were selected (the number of conditions ranged from 8 to 48), and none of the studies standardised their prevalence rate to a reference population. The crude prevalence rates of multimorbidity ranged from 16.3 to 89.4% for different age groups [16–21]. We compared the findings from Quah et al. [19] which targeted older adults age 65 years and over as it was the only study that was conducted in the primary care setting. The mean age and standard deviation of the participants was 73.9 (±6.5) years old which was comparable to the mean age of this study which was 73.5 (±7.0) years old (Table 1). The authors reported that the prevalence of multimorbidity was 89.4% which was higher than that found in this study - MM2+ CDMP was 72.7% and MM2+ Fortin was 77.5% (Table 3). This difference could be attributed to the difference in the conditions used to define multimorbidity and the data collection methods. Quah et al. [19] used interviewer-administered questionnaire while this study used electronic medical records. Several studies have reported variable concordance rates between questionnaire and electronic medical records [22, 23].

Our study found a higher prevalence rate of multimorbidity in males than females. However, the literature reported inconsistent differences in the prevalence rates between the sexes. These differences could be associated with the population source and the types of conditions studied. For example, Fortin et al. reported that more females than males were found with multimorbidity in the general population whereas the contrary was found in the practice-based population [4]. Schafer et al. reported females seemed to be more vulnerable to anxiety, depression, somatoform disorders, and pain-related morbidity while males appeared to be more vulnerable to cardiovascular and metabolic diseases [24].

Our study was based on a practice-based population; the distribution of single chronic conditions by prevalence rates (> 10%) in both multimorbidity lists were dominated by three cardiometabolic conditions (e.g. hyperlipidaemia, hypertension, diabetes) and one degenerative-related condition e.g., arthritis and/or rheumatoid arthritis. Psychological disorders (e.g., depression or anxiety) accounted for less than 2 % of all chronic conditions (Table 2). The low prevalence of psychological disorders in our study contrasted with other prevalence studies. Within the Canadian population, Ryan et al. [25] found that mood disorders were among the five most prevalent conditions in every age group. Within the Portuguese population, Prazeres et al. [26] found that depressive disorder was the third most prevalent condition. A likely reason for the low prevalence of psychological disorder was the social stigma associated with these conditions resulting in the decreased help-seeking behaviour by the local population [27].

Singapore is a multi-ethnic country which comprises of three main ethnic groups. However, the impact of ethnicity on prevalence of multimorbidity remains relatively unexplored. One study by Picco et al. reported that the crude prevalence rate of multimorbidity in Indians was higher than the Chinese and Malay [17]. This differed from our findings where the standardized prevalence rate of multimorbidity using the same cut-off threshold of two or more conditions was the highest in Chinese followed by Indians and Malay. Further studies to explore the patterns of multimorbidity among the different ethnic groups should be done.

### Comparison between MM2+ and MM3+ cut-off thresholds, and CDMP and Fortin lists

Using MM3+ as the cut-off identified a smaller number of patients with higher needs compared to MM2+ which was consistent for both lists and also consistent with the findings from a systematic review [3]. The Fortin list generated a higher prevalence rate of multimorbidity compared to the CDMP list in the same primary care population group. As well, the Fortin list seemed conceptually better suited for measuring multimorbidity in primary care. It reflected disease categories rather than single conditions and was more sensitive to capturing the full breadth of multimorbidity across the ages when compared to the CDMP list. Moreover, the Fortin list of conditions also covered all of the non-communicable diseases listed in the global burden of disease list for leading causes of disability-adjusted life year in Singapore (Additional file 3). On this basis, our study may lead to preferring Fortin’s list for international comparative studies, but further studies will need to be conducted to confirm our tentative conclusion that it was indeed a more suitable operational definition of multimorbidity in the Singapore primary care setting.

### Strengths and limitations

The main limitation of the current study was that the prevalence rates reported may be underestimated for the following reasons. Firstly, the study only provided a snapshot over a one-year period. Secondly, there may be under-reporting of chronic conditions in the electronic medical records especially for patients who seek treatment outside the polyclinics who may not have their other medical conditions recorded in the electronic medical records within the polyclinic system. Thirdly, mental health conditions were probably under-reported as we found that the prevalence was very low [27].

The major strength of the study was the rigorous mapping of ICD-10 diagnostic codes available in the primary care EMR to two multimorbidity lists (CDMP and Fortin) with two operational definitions of multimorbidity (MM2+ and MM3+).

## Conclusions

In conclusion, this is the first study describing the prevalence of multimorbidity using a large electronic medical record database in a Singapore primary care setting. We compared the prevalence of multimorbidity using two different lists of chronic conditions to measure multimorbidity. The crude prevalence rate of multimorbidity with a cut-off threshold of three conditions was 23.7 and 17.7% based on Fortin list and CDMP list respectively. The standardised prevalence rate was 17.2% (CI 17.2, 17.3) and 12.4% (CI 12.3, 12.5) based on Fortin list and CDMP list respectively. We determined that the prevalence rate of multimorbidity increased with age and that males had higher prevalence rate of multimorbidity compared to females. We also reported differences in the standardised prevalence rates of multimorbidity between the different ethnic groups.

We propose using three or more chronic conditions for defining multimorbidity as the higher cut-off threshold identified a smaller number of patients with higher needs compared to using two or more chronic conditions. Our study results also leaned towards using Fortin’s list as it was more sensitive to capturing the full breadth of multimorbidity across the ages when compared to the CDMP list in the primary care setting in Singapore.

## Supplementary Information

**Additional file 1.** CDMP List of Conditions.

**Additional file 2.** Fortin List of Conditions.

**Additional file 3.** Global Burden of Disease List vs Fortin List of Conditions.

## Data Availability

*The dataset and personal health data generated and/or analysed during the current study are not publicly available due to* the Data Protection Act Commission Singapore - Advisory Guidelines for the Healthcare Sector and legal and ethical restrictions related to data privacy protection but *are available from the corresponding author on reasonable request.*

## Abbreviations

ASE SPR: Age-stratified, sex-and-ethnicity standardized prevalence rate
CDMP: Chronic Disease Management Programme
CI: Confidence interval
COPD: Chronic obstructive pulmonary disease
CPR: Crude prevalence rate
EAS SPR: Ethnicity-stratified, age-and-sex standardized prevalence rate
MM: Multimorbidity
MM2+: Definition of multimorbidity as having 2 or more chronic conditions
MM3+: Definition of multimorbidity as having 3 or more chronic conditions
CDMP MM2+: Operational definition of multimorbidity as having 2 or more chronic conditions according to the CDMP list of chronic conditions
CDMP MM3+: Operational definition of multimorbidity as having 3 or more chronic conditions according to the CDMP list of chronic conditions
Fortin MM2+: Operational definition of multimorbidity as having 2 or more chronic conditions according to the list of chronic conditions in Fortin et al.
Fortin MM3+: Operational definition of multimorbidity as having 3 or more chronic conditions according to the list of chronic conditions in Fortin et al.
ICD-10: International Classification of Diseases, Tenth Revision
ICPC-2: International Classification of Primary Care, Second edition
MOH: Ministry of Health of Singapore
NHGP: National Healthcare Group Polyclinics
SAE SPR: Sex-stratified, age-and-ethnicity standardized prevalence rate
SD: Standard deviation
