# Supplementary material for: The prevalence of multimorbidity in primary care: a comparison of two definitions of multimorbidity with two different lists of chronic conditions in Singapore
Source: BMC Public Health. 2021 Jul 16;21:1409. doi: 10.1186/s12889-021-11464-7 (PMC8283957; doi:10.1186/s12889-021-11464-7)
Supplement: Supplementary file 1 — Additional file 1. CDMP List of Conditions. [file 12889_2021_11464_MOESM1_ESM.docx]

**Appendix 1A – CDMP^a^ List of Conditions**

| **S/No** | **Category of Condition** | **ICD10^c^ Code & Description** |
| --- | --- | --- |
| 1 | Diabetes | E10.9 (Type 1 diabetes mellitus without complication) |
|  |  | E11.9 (Type 2 diabetes mellitus without complication) |
|  |  | E14.2 (Diabetes mellitus with incipient diabetic nephropathy) |
|  |  | E14.3 (Diabetes Mellitus with retinopathy) |
|  |  | E14.31 (Unspecified diabetes mellitus with background retinopathy) |
|  |  | E14.64 (Unspecified diabetes mellitus with hypoglycaemia) |
|  |  | E14.73 (Unspecified diabetes mellitus with foot ulcer due to multiple causes) |
| 2 | Hypertension | I10 (Essential (primary) hypertension) |
| 3 | Lipids | E78.5 (Hyperlipidaemia, unspecified) |
| 4 | Stroke | I64 (Stroke, not specified as haemorrhage or infarction) |
| 5 | Asthma | J45.9 (Asthma, unspecified) |
| 6 | COPD^b^ | J44.9 (Chronic obstructive pulmonary disease, unspecified) |
| 7 | Chronic Kidney Disease | N18.9 (Chronic kidney disease, unspecified) |
| 8 | Osteoporosis | M81.99 (Other osteoporosis, site unspecified) |
| 9 | Rheumatoid Arthritis | M06.99 (Rheumatoid arthritis, unspecified, site unspecified) |
| 10 | Osteoarthritis | M15.9 Osteoarthritis (OA) - Generalised) |
| 11 | Major Depression | F32.20 (Severe depressive episode without psychotic symptoms, not specified as arising in the postnatal period) |
| 12 | Anxiety | F41.1 (ANXIETY DISORDER, UNSPECIFIED) |
| 13 | Dementia | F03 (Unspecified dementia) |
| 14 | Benign Prostate Hypertrophy | N40 (Hyperplasia of prostate) |
| 15 | Parkinson's | G20 (Parkinson's disease) |
| 16 | Epilepsy | G40.90 (Epilepsy, unspecified, without mention of intractable epilepsy) |
| 17 | Psoriasis | L40.8 (Other psoriasis) |
| 18 | Schizophrenia | F20.9 (Schizophrenia, unspecified) |
| 19 | Bipolar Disorder | F31.9 (Bipolar affective disorder, unspecified) |
| 20 | Ischaemic Heart Disease | I25.9 (Chronic ischaemic heart disease, unspecified) |

^a^ CDMP – Chronic Disease Management Program; ^b^ COPD – Chronic obstructive pulmonary disorder; ^c^ ICD-10 - International Statistical Classification of Diseases and Related Health Problems revision 10
