# Supplementary material for: The prevalence of multimorbidity in primary care: a comparison of two definitions of multimorbidity with two different lists of chronic conditions in Singapore
Source: BMC Public Health. 2021 Jul 16;21:1409. doi: 10.1186/s12889-021-11464-7 (PMC8283957; doi:10.1186/s12889-021-11464-7)
Supplement: Supplementary file 2 — Additional file 2. Fortin List of Conditions. [file 12889_2021_11464_MOESM2_ESM.docx]

**Appendix 1B – Fortin List of Conditions**

| **S/No** | **Category of Condition** | **ICD10^d^ Code & Description** |
| --- | --- | --- |
| 1 | Any cancer in the last 5 years | C80 (Malignant neoplasm without specification of site) |
| 2 | Thyroid disorder | E03.9 (Hypothyroidism, unspecified) |
|  |  | E05.9 (Thyrotoxicosis, unspecified) |
| 3 | Diabetes | E10.9 (Type 1 diabetes mellitus without complication) |
|  |  | E11.9 (Type 2 diabetes mellitus without complication) |
|  |  | E14.2 (Diabetes mellitus with incipient diabetic nephropathy) |
|  |  | E14.64 (Unspecified diabetes mellitus with hypoglycaemia) |
|  |  | E14.73 (Unspecified diabetes mellitus with foot ulcer due to multiple causes) |
| 4 | Obesity | E66.9 (Obesity, unspecified) |
| 5 | Hyperlipidaemia | E78.5 (Hyperlipidaemia, unspecified) |
| 6 | Dementia or Alzheimer's disease | F03 (Unspecified dementia) |
| 7 | Depression or anxiety | F32.20 (Severe depressive episode without psychotic symptoms, not specified as arising in the postnatal period) |
|  |  | F32.90 (Depressive episode, unspecified, not specified as arising in the postnatal period) |
|  |  | F41.1 (Anxiety disorder, unspecified) |
| 8 | Hypertension  (high blood pressure) | I10 (Essential (primary) hypertension) |
| 9 | Cardiovascular disease  (angina, AF^a^, MI^c^, poor circulation of lower limbs) | I25.9 (Chronic ischaemic heart disease, unspecified) |
|  |  | I48 (Atrial fibrillation and flutter) |
|  |  | I70.20 (Atherosclerosis of arteries of extremities, unspecified) |
|  |  | I73.9 (Peripheral vascular disease, unspecified) |
| 10 | Heart failure  (including valve problems or replacement) | I50.0 (Congestive heart failure) |
|  |  | I51.9 (Heart disease, unspecified) |
| 11 | Stroke and TIA^e^ | G45.9 (Transient cerebral ischaemic attack, unspecified) |
|  |  | I64 (Stroke, not specified as haemorrhage or infarction) |
| 12 | Asthma, COPD^b^, or  chronic bronchitis | J44.9 (Chronic Obstructive Pulmonary Disease, Unspecified) |
|  |  | J45.9 (Asthma, unspecified) |
| 13 | Stomach problem  (reflux, heartburn, or gastric ulcer) | K21.9 (Gastro-oesophageal reflux disease without oesophagitis) |
|  |  | K27.9 (Peptic ulcer, unspecified as acute or chronic, without haemorrhage or perforation) |
| 14 | Colon problem  (irritable bowel) | K58.9 (Irritable bowel syndrome without diarrhoea) |
| 15 | Chronic hepatitis | K76.9 (Liver disease, unspecified) |
|  |  | Z22.51 (Carrier of viral hepatitis B) |
| 16 | Arthritis &/or  rheumatoid arthritis | M06.99 (Rheumatoid arthritis, unspecified, site unspecified) |
|  |  | M15.9 (Osteoarthritis (OA) - Generalised) |
|  |  | M19.99 (Arthritis, Unspecified, Site Unspecified) |
| 17 | Osteoporosis | M81.99 (Other osteoporosis, site unspecified) |
| 18 | Kidney disease or failure | N03.9 (Unspecified nephritic syndrome, unspecified) |
|  |  | N18.9 (Chronic kidney disease, unspecified) |
| 19 | Chronic urinary problem | N40 (Hyperplasia of prostate) |
| 20 | Chronic musculoskeletal condition causing pain or limitation | No matching ICD code |

^a^ AF – Atrial fibrillation; ^b^ COPD – Chronic obstructive pulmonary disorder; ^c^ MI – Myocardial infarction; ^d^ ICD-10 - International Statistical Classification of Diseases and Related Health Problems revision 10; ^e^ TIA – Transient ischaemic attack
