# Supplementary material for: The prevalence of multimorbidity in primary care: a comparison of two definitions of multimorbidity with two different lists of chronic conditions in Singapore
Source: BMC Public Health. 2021 Jul 16;21:1409. doi: 10.1186/s12889-021-11464-7 (PMC8283957; doi:10.1186/s12889-021-11464-7)
Supplement: Supplementary file 3 — Additional file 3. Global Burden of Disease List vs Fortin List of Conditions. [file 12889_2021_11464_MOESM3_ESM.docx]

**Appendix 2 – Global Burden of Disease List vs Fortin List of Conditions**

| **S/N** | **Global burden of disease list*** | **Fortin list of conditions** |
| --- | --- | --- |
| 1 | Cardiovascular diseases | Cardiovascular disease (angina, AF, MI, poor circulation of lower limbs) |
|  |  | Heart failure  (including valve problems or replacement) |
|  |  | Hypertension (high blood pressure) |
|  |  | Stroke and TIA |
| 2 | Cancers | Any cancer in the last 5 years |
| 3 | Musculoskeletal disorders | Chronic musculoskeletal condition causing pain or limitation |
|  |  | Osteoporosis |
|  |  | Arthritis &/or rheumatoid arthritis |
| 4 | Mental disorders | Depression or anxiety |
| 5 | Neurological disorders | Dementia or Alzheimer’s disease |
| 6 | Other non-communicable diseases | Chronic urinary problem |
|  |  | Thyroid disorder |
|  |  | Chronic hepatitis |
|  |  | Obesity |
| 7 | Diabetes and kidney diseases | Diabetes |
|  |  | Hyperlipidaemia |
|  |  | Kidney disease or failure |
| 8 | Chronic respiratory diseases | Asthma, COPD, or chronic bronchitis |
| 9 | Digestive diseases | Stomach problem  (reflux, heartburn, or gastric ulcer) |
|  |  | Colon problem (irritable bowel) |

*Unintentional injuries, respiratory infections and tuberculosis, sense organ diseases, skin and subcutaneous diseases, maternal and neonatal disorders, self-harm and interpersonal violence, transport injuries, substance use disorders, nutritional deficiencies, other infectious diseases, HIV/AIDS and sexually transmitted infections, enteric infections and neglected tropical diseases and malaria are not covered by Fortin list of conditions.
